# Maternal separation with early weaning: a novel mouse model of early life neglect

**DOI:** 10.1186/1471-2202-11-123

**Published:** 2010-09-29

**Authors:** Elizabeth D George, Kelly A Bordner, Hani M Elwafi, Arthur A Simen

**Affiliations:** 1 Yale University School of Medicine, Department of Psychiatry, New Haven, CT, 06511, USA

## Abstract

**Background:**

Childhood adversity is associated with increased risk for mood, anxiety, impulse control, and substance disorders. Although genetic and environmental factors contribute to the development of such disorders, the neurobiological mechanisms involved are poorly understood. A reliable mouse model of early life adversity leading to lasting behavioral changes would facilitate progress in elucidating the molecular mechanisms underlying these adverse effects. Maternal separation is a commonly used model of early life neglect, but has led to inconsistent results in the mouse.

**Results:**

In an effort to develop a mouse model of early life neglect with long-lasting behavioral effects in C57BL/6 mice, we designed a new maternal separation paradigm that we call Maternal Separation with Early Weaning (MSEW). We tested the effects of MSEW on C57BL/6 mice as well as the genetically distinct DBA/2 strain and found significant MSEW effects on several behavioral tasks (i.e., the open field, elevated plus maze, and forced swim test) when assessed more than two months following the MSEW procedure. Our findings are consistent with MSEW causing effects within multiple behavioral domains in both strains, and suggest increased anxiety, hyperactivity, and behavioral despair in the MSEW offspring. Analysis of pup weights and metabolic parameters showed no evidence for malnutrition in the MSEW pups. Additionally, strain differences in many of the behavioral tests suggest a role for genetic factors in the response to early life neglect.

**Conclusions:**

These results suggest that MSEW may serve as a useful model to examine the complex behavioral abnormalities often apparent in individuals with histories of early life neglect, and may lead to greater understanding of these later life outcomes and offer insight into novel therapeutic strategies.

## Background

Childhood adversity, in the form of abuse and neglect, is prevalent throughout the world and poses a significant public health problem. In the US, 24.4 out of every 1000 children will be abused or neglected within the first year of life, and neglect is far more frequent than abuse [1]. Many individuals exposed to early life adversity go on to develop a variety of behavioral and psychiatric problems that persist well into adulthood, including both "internalizing disorders", such as depression [2,3] and anxiety [4], and "externalizing disorders", such as drug and alcohol abuse [5-7], attention deficit hyperactivity disorder [8], and delinquency [9-11]. Not all victims of abuse and neglect will go on to suffer adverse effects, however, suggesting that other factors may modulate the consequences of early life experience. Indeed, recent studies have proposed a role for genetic factors in the establishment of behavioral effects of early life adversity [12,13]. Unfortunately, the underlying molecular mechanisms of the consequences of early life neglect remain largely unknown.

The use of a reliable animal model of early life neglect would be an invaluable tool for understanding the potential mechanisms resulting in ensuing behavioral and neurobiological changes. Since the C57Bl/6 mouse genome has been sequenced and well annotated and numerous knockout and transgenic lines are available on this background, a mouse model using this strain would allow for in depth molecular analyses and, thus, would be ideal. Although maternal separation is often used as a model of early life neglect, close examination of this literature in both mice and rats yields mixed results. Examples in which experimental procedures have been successful in producing reliable behavioral effects in the offspring are plentiful, however, the majority of these cases examine rats (see [14,15] for reviews; but also see [16,17]). For instance, the standard 3 h of maternal separation of rat pups results in later alterations in hypothalamic-pituitary-adrenal axis (HPA) functioning [18-26], along with perturbations in several behavioral domains including fearfulness [27,16,30], attention [31,16], and anxiety [32-35,26]. The same 3 h period of separation in mice, however, often leads to an increase in maternal care potentially lessening the effects of separation itself [36,37]. Indeed, studies examining the consequences of early maternal separation in mice have concluded that protocols similar to those used in rats are either without effect or fail to produce a reliable phenotype [36-38].

The lack of consistency with regard to maternal separation in mice seems to be driven by at least two components: both the duration of separation and the quality of maternal care provided to the pups upon being returned to the nest. Additionally, the developmental period in which pups are separated radically alters the ensuing behavioral and physiological phenotypes, and consequences of early maternal separation can be altered or even masked by later periods of deprivation [39,40]. Adding to the complexity of the problem, results of early maternal deprivation in mice also differ tremendously depending upon the genetic strain under examination [41,42,36]. Clearly, modifications of existing protocols must be made in order to fully examine the consequences of early maternal separation in mice.

For these reasons, we devised a method of maternal separation that combines several published protocols in order to increase the likelihood of observing a reliable behavioral phenotype while minimizing fatalities to the developing offspring. Our method, referred to as Maternal Separation with Early Weaning (MSEW), includes separation periods that occur over a broad range of postnatal ages and an additional component of early weaning. Our model uses separation periods that are far longer than those typically reported in mice, however, the procedure is performed in a manner that does not compromise viability of the offspring. Additionally, pups are weaned from the dam at an earlier age, when behavioral development permits independent feeding, further limiting maternal contact. As described here, MSEW is a novel paradigm with excellent face validity that allows for in depth examination of the behavioral and neurobiological effects of maternal separation in mice, and will aid in the understanding of the molecular mechanisms driving these outcomes.

## Results

### Study Design

Mouse pups derived from experimentally naïve C57BL/6J (B6) and DBA/2J (D2) mice (Jackson Laboratories, Bar Harbor, ME) were used as experimental subjects. Our primary interest was to develop a model that would be effective in the B6 strain, and the genetically distinct and differentially stress vulnerable D2 strain was used for comparison. For breeding, a single male and 3-4 females were group-housed in plastic cages containing dried corn husk bedding and a cotton nestlet. Pregnant females were individually housed and observed daily for parturition, deemed as postnatal day (PD) 0. Upon discovery, the entire litter was randomized to either control or MSEW conditions. With the exception of body weights obtained on PDs 10 and 17 and a single cage change, control litters were left undisturbed and weaned on PD 23, the usual weaning date at our facility. In contrast, MSEW litters underwent Maternal Separation with Early Weaning *(see below)*. On PD 23, pups from both conditions were separated by sex, housed with up to four littermates, and left undisturbed except for routine cage changes every two weeks and periodic weight assessments until the start of behavioral testing (PD 65). All animals were maintained in a temperature controlled environment (22°C), on a 12:12 light-dark cycle with lights on at 0700 hours and both food and water available *ad libitum *(Harlan standard rodent chow). Mice were maintained and treated in accordance with guidelines set forth by the National Academies of Science [43] and procedures approved by the Yale University Institutional Animal Care and Use Committee (protocol 2008-10975).

### Development of the Maternal Separation with Early Weaning Model

We initially conducted a pilot study to determine whether 3 h maternal separation has long-lasting effects on behavior as determined by the elevated plus maze and forced swim test. We randomized 14 litters of C57BL/6J mice to 3 h per day of maternal separation between PD 2 and 17 and control conditions. A total of 80 male mice from the 14 litters were subsequently behaviorally tested and data were analyzed as described in detail below (see Behavioral Testing). Analysis of elevated plus maze behavior revealed no significant effects of maternal separation (p = 0.9874). Similarly, time spent immobile on the forced swim test showed no significant effect of maternal separation (p = 0.5554). We then generated three additional litters of mice that were subjected to 3 h maternal separation between PD2 and 17 and were subsequently weaned at PD17 (early weaning). Early weaning was added to the maternal separation in an effort to reduce any potential for compensatory maternal care after maternal separation had ended but prior to weaning and has been shown to cause neuroendocrine alterations and increased anxiety-like and aggressive behavior in mice [44-46]. Although previous studies have utilized weaning at PD14 (e.g. [45]), we were unable to wean our animals this early because of veterinary concerns. Analysis of time spent immobile on the forced swim test revealed a trend toward more immobility as compared to the 3 h maternal separation only condition (p = 0.0919). Analysis of time spent on the open arms of the elevated plus maze revealed no significant difference from 3 h maternal separation with weaning at PD23 (p = 0.3528).

Given the trend toward greater immobility on the forced swim test after adding early weaning to 3 h of maternal separation we hypothesized that further reductions in time spent with the dams during the daily separation period would further potentiate the effects of the manipulation. This hypothesis was motivated by the fact that Milstein et al. [36] observed an increase in maternal care after maternal separation in four of the five strains examined, including B6 animals, which may have negated the effects of maternal separation. We predicted that with sufficient separation from the dam such compensation would no longer be effective. Our primary goal was to develop a model that would be effective in B6 mice. The B6 strain is ideal for molecular analysis because it is the only mouse strain with a fully sequenced genome, and transgenic and knockout lines are readily available on this background, making it ideal for genomic analysis. We also included D2 mice for comparison because of the differential stress sensitivity of this strain as compared to B6 mice. As discussed in more detail in the Discussion section, D2 and B6 mice have often been observed to differ with regard to both basal and stress-induced levels of anxiety, although these differences have varied across studies. For example, D2 mice have been reported to be more exploratory and less anxious than B6 mice [47,48]. However, D2 mice have been found to be more anxious in dark-light testing and on the elevated plus maze, and appear to be more vulnerable to the effects of individual housing than B6 mice [49]. We therefore designed a new model of maternal separation incorporating longer periods of daily separation in addition to early weaning in order to maximally limit maternal contact while ensuring the safety and viability of the offspring. The resulting model, Maternal Separation with Early Weaning (MSEW), consisted of maternal separation for 4 h per day on PDs 2-5, and 8 h per day on PDs 6-16. During periods of separation, pups were left in their home cage while their dams were relocated to new, clean cages with *ad libitum *access to food and water. Pups remained with their littermates throughout the MSEW procedure, and cages were kept over a heating blanket to maintain a constant temperature within the cage (32-34°C) and aid in the maintenance of thermoregulation. MSEW pups were weaned from the dam on PD 17 at which point they were given moistened chow and checked daily for signs of dehydration or distress. A total of 63 animals from 11 B6 litters and 57 animals from 11 D2 litters in Cohort 1, and 35 animals from 5 B6 litters in Cohort 2, underwent the MSEW procedure. B6 animals only were used for Cohort 2 because of our particular interest in this strain.

Due to the process by which we randomly designated entire litters as control or MSEW at birth, we assessed litter size and sex ratio to ensure that there were no significant differences between treatment groups. We found no effects of MSEW, strain, or their interaction on litter size (all p > 0.05). In terms of the male to female ratio, there were again no effects of MSEW, strain, or their interaction (all p > 0.05). Additionally, no pups were lost due to MSEW; although a few deaths were observed immediately post-partum, there were no differences between control and MSEW groups and survival rate at the time of testing was not associated with treatment condition for either strain (all p > 0.05).

### Effects of MSEW

#### Body Weight and Serum Analyses

For assessment of pup viability during the MSEW procedure, body weight measurements were taken at several time points throughout the course of development, and some control and MSEW mice were sacrificed on PD 10 and PD 17 for serum metabolic analyses. Only male pups were weighed and subjected to metabolic analysis. Pups were rapidly decapitated and trunk blood was collected, stored on ice to allow for coagulation, and centrifuged for 10 min (10000rpm) for the collection of serum. Beta-hydroxybutyrate, non-esterified fatty acid (NEFA), and glucose levels were assessed by the Yale Mouse Metabolic Phenotyping Center. Corticosterone (CORT) levels were measured using a commercially available enzyme immunoassay (Assay Designs, Inc), according to manufacturer's instructions.

**Figure 1:**
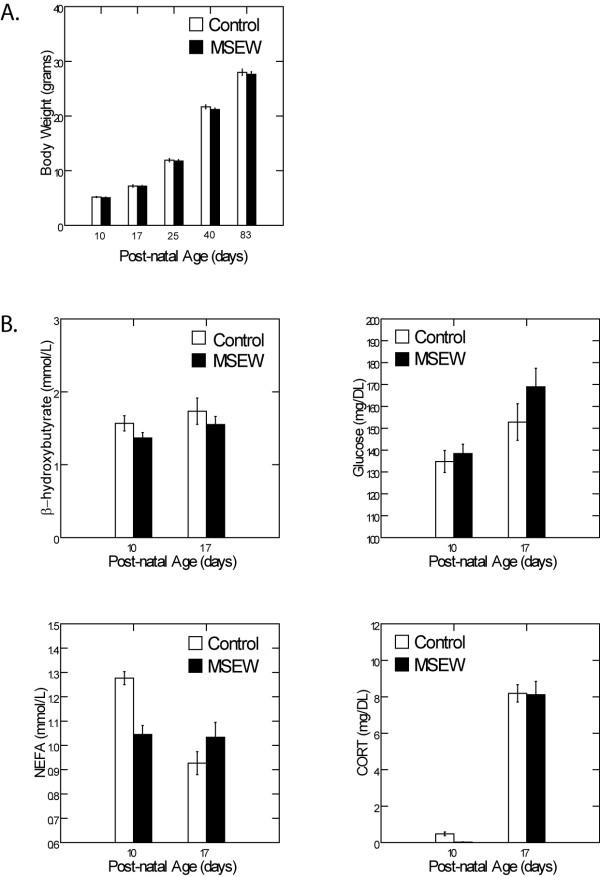
**Body Weight and Metabolic State**. MSEW has no effect on body weight throughout development (**A**). Serum levels of beta-hydroxybutyrate (**B**; top left), glucose (**B**; top right), and corticosterone (**B**; bottom right) are not affected by MSEW at PD 10 or 17. Serum levels of non-esterified fatty acids (**B**; bottom left) are affected by MSEW at PD10 (p < 0.005) but not PD17.

Analysis of body weights revealed no significant main effect of MSEW on PDs 10, 17, 25, 40, or 83 (Fig. 1A; all p > 0.05). While there was a significant main effect of age on body weight (F(4,258) = 4521.8, p < 0.0001), there was no age × MSEW interaction (p > 0.05). Additionally, no significant main effect of MSEW was found on serum levels of beta-hydroxybutyrate, glucose, NEFA, or CORT on PDs 10 or 17 (Fig. 1B; all p > 0.05). While there was no effect of age on beta-hydroxybutyrate, we did find a significant main effect of age on glucose, NEFA, and CORT (all p < 0.001). We found no age × MSEW interaction effect on levels of beta-hydroxybutyrate, glucose, or CORT, however, we did find an age × MSEW effect on levels of NEFA (F(1,29) = 15.06, p < 0.005). Contrasts revealed lower levels of NEFA for MSEW animals on PD10, but not PD17, compared to controls (F(1,29) = 12.77, p < 0.005; Fig. 1B, bottom left).

#### Behavioral Testing

To assess the long lasting effects of MSEW, a total of 73 male mice (35 MSEW, 38 Control) from 26 B6 litters and 55 male mice (29 MSEW, 26 Control) from 22 D2 litters were behaviorally tested in Cohort 1. As noted above, we were particularly interested in developing a model that is effective in B6 mice. An additional 39 male mice (20 MSEW, 19 Control) from 11 B6 litters were tested in Cohort 2. Mice were between 65 and 80 days of age during behavioral testing, and all mice underwent all behavioral assessments. Because mice were group-randomized by litter, all data presented here were analyzed using mixed-effects linear models [50,51] with MSEW, strain, and the MSEW × strain interaction as fixed effects and litter nested within treatment as a random effect [52]. Additionally, as noted below, some data were binned in 5 min intervals and analyzed in a repeated-measures fashion, with MSEW, strain, time, and all interactions as fixed effects, and mouse and litter as random effects. For all of the mixed effects models, we conducted contrasts to interpret interaction effects when interactions were statistically significant. For each of the behavioral tests, initial models included litter size as a fixed effect; no main effects or interaction effects were noted for this variable and it was therefore dropped from subsequent analyses.

#### Open Field (OF)

Mice were placed in the center of a circular white plastic open field (diameter 55 cm) with an opaque floor and 30 cm opaque walls. In contrast to a square arena, the circular open field allows for unambiguous assessment of distance from both the center and sides of the chamber [53]. The arena was placed in the center of a dimly lit room, devoid of any obvious visual cues, and was lit either with diffuse muted lighting (Cohort 1) or a single lamp placed directly above the center (Cohort 2). A camera was suspended 3 ft above the middle of the arena and each mouse was video recorded during the exploration period. Testing of Cohort 1 was performed for 30 min, and replications with Cohort 2 were conducted using an abbreviated period of 15 min, with an additional 10 min test on Day 2. Videos were scored offline using software written by the authors to determine position in the open field at each time point, and these data were was used to calculate speed, distance from the center, and distance travelled in 5 min intervals over the course of testing.

**Figure 2:**
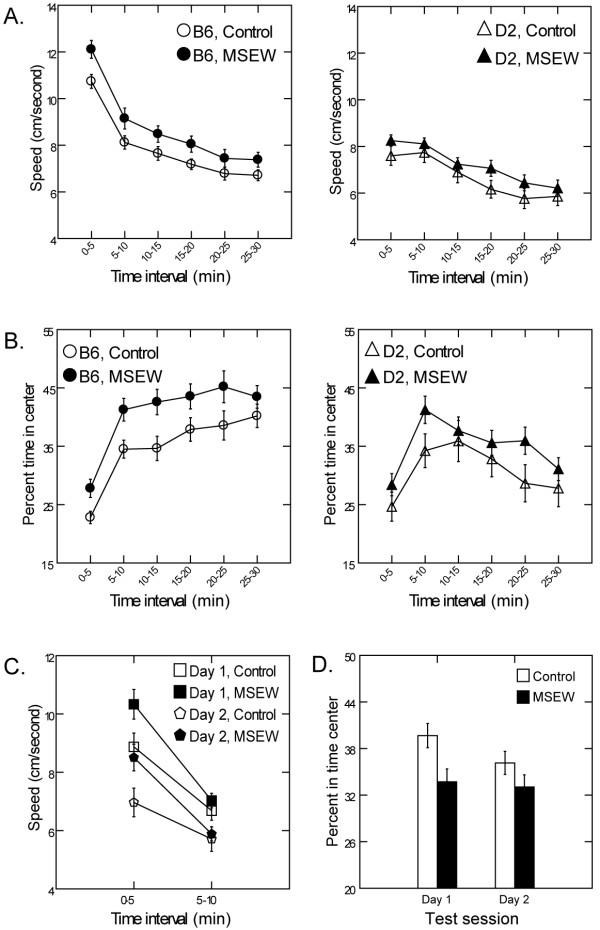
**Open Field Behavior**. Effects of MSEW on open field behavior in 5 min time bins for Cohort 1 (**A**, **B**) and Cohort 2 (**C**, **D**) mice. In Cohort 1, MSEW and B6 animals moved faster (**A**; MSEW p < 0.05; B6 p < 0.0005) and spent more time in the center of the arena (**B**; MSEW p < 0.01; B6 p < 0.001) than control and D2 mice, respectively. In Cohort 2, MSEW animals moved faster during the first 5 min of testing (**C**; p < 0.005) and spent less time in the center of the arena (**D**; p < 0.05) than controls.

Analysis of open field behavior in Cohort 1 revealed significant main effects of MSEW and strain on speed, with MSEW and B6 mice moving faster and thus covering more area than controls (F(1,41) = 4.13, p < 0.05) and D2 animals (F(1,649) = 13.93), p < 0.0005), respectively (Fig. 2A). We also found a significant main effect of time and a time × strain interaction on speed; while all animals showed a decrease in speed as a function of time (F(5,649) = 65.11, p < 0.0001), B6 mice showed a more rapid decline across the test session. Analysis of time spent in the middle of the arena also revealed main effects of MSEW and strain. MSEW and B6 animals spent a greater percent of time in the center of the open field than controls (F(1,41) = 8.62, p < 0.01) and D2 mice (F(1,649) = 11.97, p < 0.001), respectively (Fig. 2B). Additionally, there was a main effect of time, and a more complicated strain × time interaction, wherein time spent in the center increased then decreased for D2 mice, but continuously increased for B6 animals.

We sought to determine whether the elevated locomotor speed observed in the MSEW mice was due to the fact that the MSEW mice spent more time in the central portion of the open field than control mice, since mice may cross the center of the open field more rapidly than other portions of the open field. We rescored the open field data for each animal by calculating locomotor speed within concentric circles of 0-25%, 25-50%, 50-75%, and 75-100% of the radius of the open field. We again observed a significant effect of strain and group, a significant effect of distance from the center (p < .0001) and a distance from center × strain interaction (p < .0001), but no group × distance from the center interaction effect (p = 0.9739) and no strain × group × distance from center interaction effect (p = 0.3363). Therefore, the observed effects of MSEW on locomotor speed do not appear to be due to MSEW effects on distance from the center of the open field.

We were surprised that the MSEW animals spent more rather than less time in the center of the open field than controls. Previous reports have shown that the intensity of light illuminating the open field has large effects on behavior on the test in mice [48] as well as rats [54]. We therefore hypothesized that the dim and diffuse lighting used for cohort 1 was not sufficiently aversive and we therefore increased the intensity of the light illuminating the center of the open field and tested an independent cohort of B6 mice (Cohort 2). In addition, we sought to determine whether the effects of MSEW on locomotor speed observed in Cohort 1 were due to the novelty of the open field environment or due to a primary effect on locomotor speed. We therefore tested Cohort 2 mice on two successive days in the open field.

Analysis of Cohort 2 revealed a similar, though marginally significant effect of MSEW, with MSEW mice again moving faster than controls (p = 0.06). More detailed analysis revealed a time × MSEW interaction, such that MSEW animals moved significantly faster than controls during the first, but not last, 5 min of testing (F(1,124) = 9.01, p < 0.005; Fig. 2C). In addition, we found a main effect of test day; all mice moved at a slower rate on Day 2, however, MSEW mice still moved significantly faster than controls during the first 5 min, even after prior exposure to the open field suggesting that the locomotor effects of MSEW are not solely due to the novelty of the open field. With regard to time spent in the center of the arena, there was a significant main effect of MSEW, with mice in Cohort 2 spending less time in the center after separation (F(1,8) = 7.81,p < 0.05), irrespective of test day (Fig. 2D). Therefore, as predicted, more intense illumination of the center of the open field caused MSEW mice to avoid the central portion of the open field, in contrast to what was observed with diffuse low level illumination in cohort 1.

#### Elevated Plus Maze (EPM)

Elevated plus maze testing was performed in a manner similar to that previously described [55]. Mice were given 15 min to explore a plus shaped maze, constructed of white Plexiglass according to the dimensions of most commercially available mouse mazes (e.g., San Diego Instruments, Panlab Harvard Apparatus). The maze, positioned 31.5 cm above the floor, contained two open and two closed arms, all 30 cm in length, connected by a 6 cm center square. Closed arms were surrounded by 28 cm high black walls. The maze was placed inside an opaque testing box (100 cm × 100 cm × 30 cm), which was positioned in the center of a dimly lit room devoid of any obvious visual cues. A video camera placed 3 ft above the maze acquired digital video, which was later viewed by an observer blind to experimental condition. Behavior was scored in 5 min intervals for total open and closed arm entries (defined as all four limbs entering the arm), percent open arm entries ((open arm entries/total arm entries) × 100), protected and unprotected stretch-attends, and protected and unprotected head dips (protected defined as occurring within a closed arm, and unprotected occurring on an open arm). Additionally, video tracking was performed offline using software written by the authors to determine time spent in each arm and speed at each time point.

**Figure 3:**
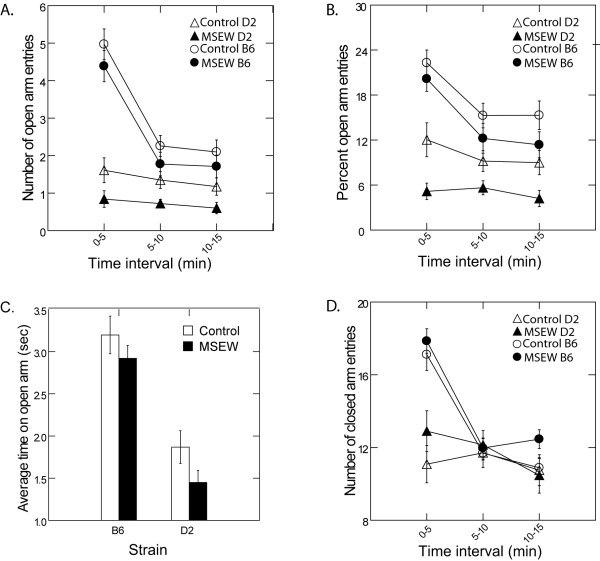
**Elevated Plus Maze Behavior**. Effects of MSEW on elevated plus maze behavior, presented in 5 min intervals. MSEW and D2 mice made fewer open arm entries (**A**; MSEW p < 0.005; D2 p < 0.0001), a smaller percent of open arm entries (**B**; MSEW p < 0.0001; D2 p < 0.0001), and spent less time on open arms per entry (**C**; MSEW p = 0.08; D2 p < 0.0001) than control and B6 animals, respectively. MSEW had no effect on closed arm entries (**D**).

Analysis of elevated plus maze behavior revealed significant main effects of MSEW and strain on open arm entries. The total number of open arm entries was significantly lower for MSEW than control animals of both strains (F(1,106) = 10.24, p < 0.005), and D2 mice, regardless of treatment, made far fewer entries than their B6 counterparts (F(1,106) = 103.94, p < 0.0001). In addition, we found that open arm entries decreased as a function of time for all animals (F(2,212) = 31.27, p < 0.0001), and there was a significant strain × time interaction, such that B6 mice showed a greater decline in number of entries across the session (F(2,212) = 21.06, p < 0.0001) (Fig. 3A). Similarly, MSEW and D2 animals made a smaller percent of open arm entries compared to controls (F(1,106) = 17.37, p < .0001), and B6 mice (F(1,106) = 77.41, p < 0.0001), respectively. Again, we observed a significant main effect of time, and a significant strain × time interaction. Specifically, all animals showed a reduction in percent open arm entries across the test (F(2,212) = 10.24, p < .0001), and this decline was more rapid in B6 mice (F(2,212) = 4.38, p < 0.05) (Fig. 3B). Analysis of total time spent on open arms revealed no main effect of MSEW, though there was a trend in average time spent in an open arm per entry, with mice spending less time per entry after MSEW (p = 0.08). We did, however, find a significant main effect of strain where B6 animals spent more time on the open arms per entry than D2 mice (F(1,81) = 49.45, p < 0.0001) (Fig. 3C). There was no effect of MSEW on speed while on an open arm (p > 0.05).

In contrast to open arm entries, analysis of closed arm entries showed a trend toward more entries by MSEW mice (p = 0.08). We again found a significant main effect of strain; D2 mice of both treatment groups made fewer arm entries than their B6 counterparts (F(1,120) = 23.92, p < 0.0001). We also found a significant main effect of time and a significant strain × time interaction, such that closed arm entries declined as a function of time for all animals (F(2,235) = 24.97, p < 0.0001), and declined more steeply for B6 mice (F(2,235) = 15.07, p < 0.0001) (Fig. 3D). Analysis of total time on closed arms again revealed no main effect of MSEW, however, we saw a significant effect of MSEW on average time spent in a closed arm per entry, with mice again spending less time per entry after MSEW (F(1,34.1) = 6.81, p < 0.05; data not shown). Additionally, analysis of speed in the closed arms revealed a trend toward an effect of MSEW, with separated mice moving faster than controls (p = 0.057).

With regard to exploratory activity, many more protected than unprotected head dips were observed (F(1,116) = 17.83, p < 0.0001). While no main effect of MSEW was apparent, we did find an effect of strain, in that B6 mice made more head dips of both types than D2 mice (F(1,116) = 120.10, p < 0.0001). We also found a significant strain × type interaction, with D2 mice making far more protected than unprotected head dips (F(1,116) = 14.27, p < 0.001), while B6 mice made an approximately equal number of each type (data not shown). Similarly, all mice made more protected than unprotected stretch-attends (F(1,116) = 711.74, p < 0.0001), and we again observed an effect of strain, such that B6 mice made more stretch attends of both types than their D2 counterparts (F(1,116) = 59.18, p < 0.0001). In addition, we found a significant effect of MSEW on the type of stretch-attend made, where MSEW mice make more protected, and fewer unprotected stretch-attends, compared to controls (F(1,116) = 4.53, p < 0.05) (data not shown).

#### Forced Swim Test (FST)

The forced swim test was performed according to published procedures [56,57] with minor modifications (see). Mice were placed in a 4-L glass cylinder (16 cm diameter) filled to a depth of 10 cm with 25°C water. Each mouse was tested for 15 min and the cylinder was cleaned and filled with fresh water following each animal. At completion, mice were removed from the water, briefly dried, placed in a holding cage, and put under a heat lamp for 30 min before being returned to their home cage. Digital video was acquired from above and later scored in 5 min intervals by an observer blind to experimental condition. Behavior was classified as immobile (defined as the absence of movement with exception of what is necessary to keep the animal's head above water), active swim (defined as movement of all four limbs), or mild swim (defined as low frequency movement involving only one or two limbs).

**Figure 4:**
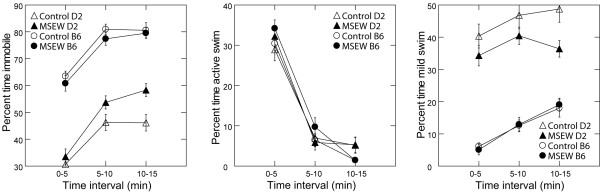
**Forced Swim Test**. Effects of MSEW on percent time spent in immobile (**left **panel), mild swim (**right **panel), and active swim (**middle **panel) states during forced swim testing, in 5 min intervals. D2-MSEW mice spent more time immobile (p < 0.05) and less time in mild swim (p < 0.05) than D2-controls. D2 mice spent less time immobile (p < 0.0001) and more time in mild swim (p < 0.0001) than B6 animals. There were no effects of MSEW or strain on active swim.

Analysis of forced swim behavior revealed no main effect of MSEW on time spent immobile, however, there was a significant strain × MSEW interaction (F(1,81) = 7.7864 p < 0.01). While B6 mice were not affected by separation, D2-MSEW mice spent significantly more time immobile compared to D2-controls (F(1,81) = 6.96, p < 0.05) (Fig. 4 left). Likewise, although we found no main effect of MSEW on mild swim, the strain × MSEW interaction approached significance (p = 0.08). Again, B6 mice were not affected by separation, while D2-MSEW mice spent significantly less time in mild swim than D2-controls (F(1,325) = 5.47, p < 0.05) (Fig.4 right). In addition, we found a main effect of strain on time spent immobile (F(1,81) = 244.4884 p < 0.0001; Fig. 4 left) and time spent in mild swim (F(1,325) = 116.8591 p < 0.0001; Fig. 4 right); regardless of treatment, D2 mice spent less time immobile and more time in mild swim compared to their B6 counterparts. There was no effect of MSEW, strain, or their interaction on active swim (all p < 0.05). Lastly, we observed a significant main effect of time on all states; all mice spent more time immobile (F(2,325) = 71.5, p < 0.0001; Fig. 4 left), more time in mild swim (F(2,325) = 16.1, p < 0.0001; Fig. 4 right), and much less time in active swim (F(2,325) = 365.8, p < 0.0001; Fig. 4 middle) following the first 5 min of the test session.

## Discussion

The current study was designed to define an experimental manipulation that would produce a robust, long lasting behavioral phenotype in B6 mice and serve as a reliable model of early childhood neglect. Previous studies have suggested that 3 h of maternal separation in mice often leads to an increase in maternal care potentially lessening the effects of separation itself [36,37]. Studies examining the consequences of maternal separation in mice have concluded that 3 h of maternal separation is either without effect or fails to produce a reliable phenotype [36-38] and our pilot studies were consistent with these findings. We hypothesized that the efficacy of the procedure would be increased by increasing the duration of the daily separation period and by weaning the animals at PD17, after the last day of maternal separation. Early weaning itself has been shown to cause neuroendocrine alterations and increased anxiety-like and aggressive behavior in mice [44-46]. However, in our pilot studies, the addition of early weaning (defined as weaning at PD17) to 3 h maternal separation was not sufficient to cause behavioral changes. It should however be noted that other studies (e.g. [45]) used weaning at PD14 whereas we weaned pups at PD17 which may have lessened the efficacy of this procedure. When we combined early weaning with extended separation periods (Maternal Separation with Early Weaning; MSEW) we observed multiple behavioral changes that persisted well into adulthood and were apparent across a variety of behavioral domains. We propose that MSEW is superior to previously used maternal separation protocols in that it produces a robust phenotype in the offspring, while being safe for pups. In addition, because MSEW is effective in B6 mice this model should prove useful for studies examining the cellular and molecular consequences of early maternal neglect.

### MSEW does not cause alterations in metabolite levels or body weight

It is reasonable to question whether the long lasting behavioral effects of MSEW are in fact due to the separation itself, or if there are other contributing factors. Admittedly, standard maternal separation times are much shorter than those employed in the current study, and may, therefore, not be as potentially detrimental to the nutritional state of the pup. In the case of MSEW, it is possible that the longer separation times would lead to nutritional deficiencies. To determine whether MSEW affected the nutritional state of pups, we analyzed body weight throughout development as well as metabolic indices of nutritional status. Importantly, analysis of body weight confirmed that there were no changes due to MSEW. Further, MSEW did not result in any nutritional deficits or altered HPA activity during the time of separation, as evidenced by serum analyses of several markers of malnutrition. Some serum metabolites, such as beta-hydroxybutyrate and NEFA, rise during periods of food restriction [58], and heightened levels can, therefore, serve as a marker of malnutrition. We did not see any effect of MSEW on serum levels of glucose or beta-hydroxybutyrate on PDs 10 or 17, or NEFA on PD17. In fact, the lone metabolic alteration during MSEW, a significant reduction in serum NEFA in PD10 MSEW pups, suggests a possible increase in overnight feeding, perhaps to account for lack of nursing during separation. The lack of effect on animal weights and serum metabolic factors suggests that MSEW does not induce a significant change in total feeding behavior. The lack of an MSEW induced increase in CORT, however, was somewhat unexpected, since extended periods of maternal separation have proven effective in eliciting robust HPA activation (e.g., [59-63]). Notably, in the current experiment, serum was collected prior to any period of separation on that day, so MSEW-induced increases in CORT were possibly dampened by the previous night's maternal care. Thought not a direct indication, this effect along with the lack of metabolic differences in the MSEW animals suggests that lasting behavioral effects were due to MSEW itself and not due to intervening factors such as nutritional status or increased HPA activity during early development.

While the developmental differences in serum corticosterone appear to be striking, attention must be paid to the day of assessment. In both the mouse and rat, a period of stress hyporesponsiveness (SHRP) is associated with blunted HPA activity and reduced basal and stress-induced CORT release (e.g., [64-67]). Developmental differences were also observed in other measures, including body weight, and several metabolic factors (e.g. glucose and NEFA). These differences, while significant, are in line with what one would expect to observe across early ontogeny and reflect normal growth and development.

### MSEW results in long lasting behavioral changes

#### Open field testing reveals hyperlocomotion and altered anxiety of MSEW animals

Mice were behaviorally characterized 2 months following completion of the MSEW procedure. The first behavioral test, the open field, is commonly used to assess emotionality in rodents using measures of locomotion and anxiety [68,69]. Perhaps the most notable behavioral effect of MSEW in the open field was a significant increase in speed, resulting in greater distances travelled over the course of testing. The increased locomotor activity observed in MSEW animals of both strains suggests a hyperactivity phenotype. This effect was both replicated in a second cohort of B6 animals and is consistent with findings from other labs in rat models of maternal separation [31]. Importantly, this finding is also similar to the effects observed clinically in adults with a history of early life adversity [8]. Though most apparent during the first 5 min of testing, the hyperactivity phenotype persisted on Day 2, indicating that it was not simply driven by exposure to a novel environment.

Measures of anxiety in the open field showed an important effect of light intensity on MSEW effects on time spent in the center of the open field. MSEW animals from Cohort 1 spent more time in the center of the arena than controls, while the opposite was true for animals from Cohort 2. During Cohort 1 testing the arena was illuminated uniformly and with a low light level, and MSEW mice spent more time in the center of the open field than controls. To better evaluate what appeared to be a reduction in anxiety, the center of the open field was more brightly lit for Cohort 2. As expected, MSEW animals from Cohort 2 exhibited increased avoidance of the center of the field, a response that represents a natural avoidance of open areas [70], and is consistent with other reports of heightened anxiety following maternal separation [32-35,26]. The reversal of the effects noted with respect to time spent in the center when the central portion was more intensely illuminated suggests that MSEW causes avoidance of the center of the open field only with sufficient light intensity. This finding is consistent with previous reports showing that the intensity of light illuminating the open field has large effects on behavior on the test in mice [48] as well as rats [54]. The increased time spent in the center of the open field in MSEW animals under low light conditions was unexpected. It is possible that at low light levels the test fails to measure anxiety-like behavior and is, instead, sensitive to other behaviors such as exploration. Alternatively, it is possible that at low stress levels MSEW mice are less avoidant of open spaces than are controls, but that the pattern reverses with higher levels of stress, possibly because of enhanced stress vulnerability in the MSEW animals. Further research will be required to distinguish between these possibilities.

#### Elevated plus maze testing confirms hyperlocomotion and suggests increased anxiety after MSEW

We used the elevated plus maze to further characterize the behavioral phenotype resulting from MSEW. Though commonly used as a test of anxiety in mice, the EPM also assesses locomotor activity, and can therefore both confirm and extend results obtained in the open field [71-73]. Behavior of MSEW mice on the EPM does, in fact, confirm the hyperactivity phenotype, as evidenced by a faster rate of movement while in closed arms. The behavior of MSEW mice on the elevated plus maze also suggests increased anxiety. Specifically, MSEW mice made fewer open arm entries, and showed a decrease in percent open arm entries and a trend toward less time spent on an open arm per entry. Like the center of a brightly lit open field, the open arms of an elevated plus maze are typically avoided due to a rodent's natural aversion to open spaces [70]. The increased avoidance of open arms and reduction in time spent on open arms per entry both point to increased anxiety in MSEW mice. Additionally, the anxiety-like phenotype is further supported by analysis of stretch-attend behaviors; while MSEW animals show a general increase in exploratory activity (i.e., increased protected stretch attends), they make far fewer unprotected stretch attends than controls. Analysis of EPM behavior, together with that in the open field, provides strong support for hyperactivity and increases in anxiety due to MSEW. Notably, these results are consistent with long lasting effects of early life adversity typically reported in both animal models (e.g., [32-34]) and clinical studies (e.g., [2,4,8,3]).

#### Forced Swim testing reveals increased depressive like symptoms

The forced swim test is a commonly used test of depressive-like behavior, where mice are forced into an inescapable situation and time spent immobile is considered a measure of behavioral despair [56,57]. Anti-depressants have been shown to alter activity, specifically leading to a reduction in time spent immobile [56,57]. Using a 15 min test, we found significant effects of time on almost all measures analyzed, consistent with previously reported observations [74]. Further, effects of MSEW were most apparent during the final 5 min interval of the test, in agreement with previous studies showing that the effects of antidepressant treatment are greater following the first 5 min of forced swim [75]. These results suggest that a longer testing time may be useful in determining small behavior changes, and may be necessary when testing mice, as previous studies have failed to find consistent effects using shorter testing intervals (e.g., [36,37]). In the present study, D2 mice spent more time immobile and less time in mild swim after MSEW, suggesting an increase in depressive-like behavior as a result of separation. In contrast, B6 mice seemed to be relatively unaffected by MSEW with regard to behavior in the forced swim test. The considerable MSEW × strain interaction that we observed suggests that MSEW-induced alterations in behavioral despair are governed by genetic factors, and warrants further investigation.

#### The Role of Strain in Response to MSEW

Although our primary goal was to develop a model that would be effective in the B6 strain, numerous B6 versus D2 strain differences emerged from this work that are of interest. Strain alone was found to have a very strong main effect on nearly all of the tests performed. Relative to B6, D2 mice stayed further away from the center of the arena and moved more slowly during the open field test. On the elevated plus maze, D2 mice made fewer arm entries, and spent more time in closed arms and less time on open arms. Overall, this pattern of activity suggests a higher level of anxiety-like behavior in the D2 strain as compared to B6 mice, a notion that supports the results of previous studies ([76-80,49] but see [81,47] for evidence to the contrary). Also consistent with several previous reports, D2 mice spent less time immobile during the forced swim test, suggestive of less depressive-like behavior at baseline ([82-84] but see [85,86] for evidence to the contrary). Notably, the forced swim test, used as an index of behavioral despair, and the open field and elevated plus maze tests, both measures of anxiety, elicit different responses in the two strains. The genetic differences between B6 and D2 mice may give insight into the mechanisms involved in depressive- versus anxiety- like responses to MSEW. It is reasonable to question whether the strain differences in response to MSEW are due to the separation procedure itself, or perhaps separation-induced alterations in maternal care. Several published reports, however, have shown that maternal care in B6 and D2 dams is roughly equivalent, both at baseline and after periods of maternal separation [36,87]. While additional studies are required to determine the molecular basis of these strain dependent effects, we believe that these differences in response to MSEW provide a means to determine genetic factors that modulate the effects of early life adversity.

## Conclusions

The MSEW procedure presented here is a novel paradigm proposed as a model of early life neglect. Previously, most commonly used methods of maternal separation have produced inconsistent results, and we believe a reliable model of maternal neglect in the B6 mouse is necessary in order to fully understand the mechanisms leading to later life psychiatric problems resulting from early life neglect. We have shown that MSEW is not associated with increases in pup mortality or morbidity, weight change, or metabolic derangements. Subsequent to MSEW, we observed alterations across several behavioral domains, most notably increases in hyperactivity, anxiety, and depressive- like symptoms. Further, the two strains tested differed in their behavioral response to MSEW on some measures analyzed, suggesting an additional genetic component to the effect of MSEW. We believe that MSEW provides an effective model of maternal neglect which has, until now, not been sufficiently demonstrated in the laboratory mouse. Further, we propose MSEW as a useful model with which to examine and understand the cellular and molecular mechanisms underlying the long lasting effects of early life neglect.

Phylogenetically, the mouse is a much simpler organism than the human and differences in response to early life adversity are to be expected between the two species. That being said, we feel that MSEW has strong face validity as a model of childhood neglect; we observed many parallels between the effects of MSEW in mice and symptoms that have been observed in victims of childhood neglect. For instance, the hyperactivity and heightened anxiety resulting from MSEW mirror the increased risk for attention deficit and hyperactivity disorder and anxiety disorders in victims of childhood maltreatment [9,10,4,8]. In addition, the behavioral despair observed in D2 animals on the forced swim test is consistent with a higher risk for depression among some adults who suffered from early life adversity [88,3].

We are unable to determine the mechanisms that mediate the long lasting effects of MSEW on behavior on the basis of the present data. However, we believe that the MSEW model will prove useful in determining these mechanisms. Conceptually, stress effects are mediated by vulnerability and protective factors which include genetic factors as well as complex gene × environment interactions. After cessation of stress these effects must be encoded in some manner to continue to affect behavior. This encoding likely includes neuroanatomical changes that are caused by early life neglect, neurodevelopmental abnormalities that arise because of the neglect, and molecular correlates of these structural changes. For example, a recent review of child abuse and neglect [89] noted that human survivors of neglect and/or abuse show decreased cerebral volumes, enlarged ventricles, alterations in white matter, alterations in cortical asymmetry, reduced hippocampal volumes, and other structural brain changes. There is also evidence for a role of epigenetic factors in mediating the lasting effects of maternal care in preclinical models [90,91] and epigenetic alterations are evident in human victims of child abuse [92]. Overall, we believe that the MSEW model will prove useful in determining the cellular and molecular mechanisms that mediate these long lasting effects of early life neglect. Such understanding may lead to better diagnostic and therapeutic strategies for victims of early childhood neglect.

## Competing interests

The authors declare that they have no competing interests.

## Authors' contributions

EDG participated in study design, performed MSEW procedure, performed behavior assessments, aided in data analysis, and participated in drafting the manuscript. KAB participated in behavioral analyses, aided in tissue and serum collection, carried out CORT assays, and participated in drafting the manuscript. HME participated in study design, carried out initial separation studies and behavioral assessments, and read and revised the manuscript. AAS conceived of the study, performed statistical analyses, participated in drafting the manuscript, and read and revised the manuscript draft. All authors read and approved the final manuscript
